# High density genetic maps of St. Augustinegrass and applications to comparative genomic analysis and QTL mapping for turf quality traits

**DOI:** 10.1186/s12870-018-1554-4

**Published:** 2018-12-12

**Authors:** Xingwang Yu, Jennifer A. Kimball, Susana R. Milla-Lewis

**Affiliations:** 1 Department of Crop and Soil Sciences, N.C. State University, Box 7620, Raleigh, NC 27695-7620 USA; 2 Department of Agronomy and Plant Genetics, University of Minnesota, St. Paul, MN 55108-6026 USA

**Keywords:** St. Augustinegrass, Linkage map, Comparative genomic analysis, QTL

## Abstract

**Background:**

St. Augustinegrass [*Stenotaphrum secundatum* (Walt.) Kuntze] is a warm-season, perennial turfgrass species well adapted for home lawns and commercial landscapes with economic and ecological value. However, a lack of genomic resources in St. Augustinegrass has hindered the full utilization of genetic variance for maximizing genetic gain and limited our understanding of the species’ evolution.

**Results:**

In this study, we constructed the first high-density linkage map for St. Augustinegrass using a genotyping by sequencing (GBS) approach. The integrated linkage map consists of 2871 single nucleotide polymorphism (SNP) and 81 simple sequence repeat (SSR) markers, spanning 1241.7 cM, with an average distance of 0.4 cM between markers, and thus represents the densest genetic map for St. Augustinegrass to date. Comparative genomic analysis revealed inter-chromosome arrangements and independent nested chromosome fusion events that occurred after St. Augustinegrass, foxtail millet, sorghum, and rice diverged from a common ancestor. Forty-eight candidate quantitative trait loci (QTL) were detected for turf quality-related traits, including overall turf quality, leaf texture, genetic color, and turf density. Three hot spot regions were identified on linkage groups LG3 and LG8, where multi-QTL for different traits overlapped. Several leaf development related genes were contained within these identified QTL regions.

**Conclusions:**

This study developed the first high-density genetic map and identified putative QTL related to turf quality, which provide valuable genetic resources for marker-assisted selection (MAS) in St. Augustinegrass.

**Electronic supplementary material:**

The online version of this article (10.1186/s12870-018-1554-4) contains supplementary material, which is available to authorized users.

## Background

St. Augustinegrass (*Stenotaphrum secundatum* [Walt.] Kuntze) is a warm-season turfgrass that is well adapted to tropical and subtropical regions of the world [1]. The grass is native to the Gulf of Mexico, the West Indies and Western Africa, and has been widely used along the Gulf Coast in the U.S., Southern Mexico, throughout the Caribbean region, South America, South Africa, Western Africa, Australia, and the South Pacific and Hawaiian Islands. St. Augustinegrass exhibits superior shade tolerance and moderately low input requirements compared to other turf species [2]. It has been a popular turfgrass in the southern United States for its broad leaf blades, and rapid stolon elongation, which makes the grass well-suited for sod production [3].

St. Augustinegrass belongs to tribe Paniceae in the subfamily Panicoideae, one of the largest subfamilies in Poaceae (grass family). This large subfamily contains many species of important economic value, including lawn grasses centipedegrass (*Eremochloa ophiuroides*) and St. Augustinegrass (*Stenotaphrum secundatum*) [4], biofuel stocks switchgrass (*Panicum virgatum*) [5] and other important crops, such as foxtail millet (*Setaria italica*), sorghum (*Sorghum bicolor*) and corn (*Zea mays*) [6]. This subfamily includes enormous morphological, physiological and cytological diversity and several basic chromosome numbers. It has been reported that *x* = 9 and *x* = 10 predominate the basic chromosome number in Panicoideae [6]. Understanding the mechanism by which chromosome numbers evolved is a key component to successfully deciphering genome evolution in the grasses. Thus, there is great interest in understanding the comparative genomics relationships among St. Augustinegrass and other members within Panicoideae.

The basic chromosome number of St. Augustinegrass is *x* = 9, with diploids (2*n* = 2*x* = 18), triploids (2*n* = 3*x* = 27), and tetraploids (2*n* = 4*x* = 36) reported [7], although aneuploid (2*n* = 28, 2*n* = 32) genotypes have also been identified more recently [3]. Molecular markers can be used to identify cultivars and characterize germplasm diversity. More importantly, molecular markers can be used to construct genetic maps, and together with phenotypic data, can be used to map genomic regions controlling traits of interest. In past years, progress has been made in the development of molecular markers and construction of linkage maps in St. Augustinegrass. Simple sequence repeats (SSR) markers have been developed and used to evaluate St. Augustinegrass [8, 9]. Recently, Kimball et al. reported the first linkage map for St. Augustinegrass with 160 SSR markers consisting of 9 linkage groups and spanning 1176.24 cM [10]. In addition, multiple QTL associated with winter survival-related traits were identified using this linkage map. However, this is a low marker density map, making it insufficient for fine mapping traits of interest. Furthermore, PCR-based markers like SSRs make the genotyping time and labor required high. In order to conduct large-scale association mapping and improve the efficiency of marker-assisted selection (MAS) in breeding efforts, the development of high-density linkage maps using new types of molecular markers such as single nucleotide polymorphism (SNP) is essential.

High-throughput sequencing technologies provide new tools for developing large numbers of SNP markers, exploring species diversity, constructing linkage maps and performing genome-wide association studies (GWAS) [11, 12]. Genotyping by sequencing (GBS), a simple highly multiplexed system for constructing reduced representation libraries for genetic analysis and genotyping, is becoming increasingly important as a cost effective and unique tool for MAS breeding in a range of plant species [13–17]. Most importantly, GBS is an excellent approach for plant breeding applications even in the absence of a reference genome [13].

High density linkage maps are valuable genetic tools for mapping quantitative trait loci (QTL) and map-based gene cloning. To fully explore the genomic evolution of St. Augustinegrass and understand the genetic mechanisms that determine turf quality, in this study we aimed to 1) construct high density linkage maps integrating SNP and SSR markers using a GBS approach; 2) conduct a comparative genomic study with other grass species and 3) identify QTL associated with turf quality traits.

## Results

### Genotype by sequencing and SNP discovery

The “pseudo-F2” mapping strategy enabled us to generate linkage maps for non-inbred species [18]. GBS libraries were constructed for the two parents (‘Raleigh’ and ‘Seville’) and 115 pesudo-F_2_ hybrids and sequenced on Illumina HiSeq 2500. A total of 236.6 million raw single-end reads were obtained from sequencing. By identifying barcodes and cut sites, low quality reads were removed and 211.5 million high quality reads were kept. The number of reads for each hybrid ranged from 0.48 million to 3.3 million, with an average of 1.6 million. The parental lines were sequenced at a relatively higher depth than the F_1_ hybrids to maximize the potential of detecting segregating SNPs in the parents. A total of 7.6 million and 6.5 million reads were obtained for ‘Raleigh’ and ‘Seville’, respectively, approximately 4.7 and 4 fold the hybrids averages (Additional file 1: Table S2).

The non-reference GBS SNP calling pipeline UNEAK was used to discover SNP markers. A total of 19,810 bi-allelic polymorphic sites were identified between the two parents. After discarding SNPs with low genotyping rates (< 90%) and significantly distorted (*P* < 0.01) segregation ratios, 2871 high quality SNPs were obtained for linkage map construction. These consisted of 1100 ‘lm x ll’, 1571 ‘nn x np’, and 200 ‘hk x hk’ type SNPs (For definition of allele scoring refer to Methods section.).

### Linkage map construction

**Fig. 1:**
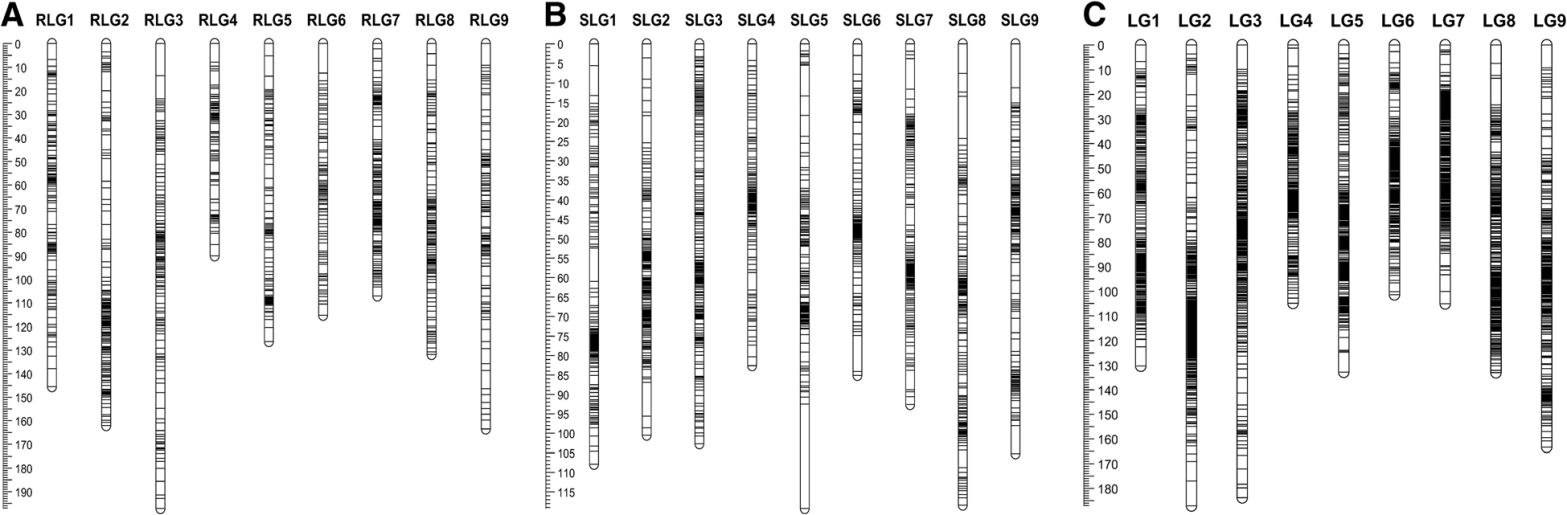
Distribution of molecular markers on parental and integrated genetic maps of St. Augustinegrass. **a** Map of parent ‘Raleigh’. **b** Map of parent ‘Seville’. **c** Integrated map from both parents. A black bar indicates a molecular maker. Linkage group number is shown on x-axis and genetic distance is shown on y-axis (cM)

Filtered markers were loaded into JoinMap 4.0 [19] for linkage map construction. Initially, ‘lm x ll’ and ‘nn x np’ type SNPs were used to construct separate linkage maps for each parent. For the linkage map of ‘Raleigh’, 1100 SNPs were mapped into nine linkage groups (LGs) (Fig. 1a), named RLG1-RLG9, which were designated based on homology between the ‘Raleigh’ map and the foxtail millet genome. The number of SNP markers on each LG varied with a maximum of 151 on RLG7 and a minimum of 78 on RLG4 (Table 1). All nine LGs spanned a total distance of 1238.7 cM, with an average distance of 1.1 cM between markers. While RLG3 was the longest with 197.2 cM, RLG4 was the shortest with 90.0 cM. The same LG number was observed on the linkage map of ‘Seville’, named SLG1-SLG9 based on homology with the genome of foxtail millet (Fig. 1b). Total length for all the ‘Seville’ LGs was 914.2 cM, with average distance 0.6 cM between markers (Table 1). For each individual LG, sizes ranged from 82.6 cM of SLG4 to 119.3 cM of SLG5. SLG3 included the most SNPs (223), while SLG6 included least (128). Compared with the ‘Raleigh’ map, the ‘Seville’ map included more SNP markers but covered a shorter length.

**Table 1:** Linkage group (LG) assignment, marker numbers, LG size and marker density in St. Augustinegrass SNP-based genetic maps

	Number of markers			Size (cM)			Average distance between markers (cM)		
Linkage group	‘Raleigh’ map	‘Seville’ map	Integrated map	‘Raleigh’ map	‘Seville’ map	Integrated map	‘Raleigh’ map	‘Seville’ map	Integrated map
LG1	132	172	337 (5)	145.5	107.9	130.3	1.1	0.6	0.4
LG2	148	189	373 (14)	162.1	100.5	187.1	1.1	0.5	0.5
LG3	142	223	390 (8)	197.2	102.7	183.8	1.4	0.5	0.5
LG4	78	150	240 (5)	90.0	82.6	104.9	1.2	0.6	0.4
LG5	108	175	326 (11)	126.3	119.3	132.8	1.2	0.7	0.4
LG6	93	128	279 (12)	115.3	85.1	101.4	1.2	0.7	0.4
LG7	151	200	374 (3)	107.1	92.6	105.1	0.7	0.5	0.3
LG8	129	164	328 (12)	131.9	118.4	133.0	1.0	0.7	0.4
LG9	119	170	305 (11)	163.5	105.2	163.3	1.4	0.6	0.5
Total	1100	1571	2952 (81)	1238.7	914.2	1241.7	1.1	0.6	0.4

^*^ Number in parentheses indicate number of SSR markers included

The ‘lm x ll’ and ‘nn x np’ type together with ‘hk x hk’ type SNP and previously identified SSR markers [10] were used to construct an integrated map for St. Augustinegrass. The integrated map consisted of 2871 SNP and 81 SSR markers, which were distributed on nine LGs (Fig. 1c). The total genetic length of the integrated map was 1241.7 cM, with an average distance of 0.4 cM between markers (Table 1). The longest LG was LG2 (187.1 cM), while LG6 was the shortest one (101.4 cM). The full information of markers in ‘Raleigh’, ‘Seville’ and integrated maps can be found in Additional file 1: Tables S3, S4 and S5. By anchoring the SSR markers with the previous linkage map constructed by Kimball et al. [10], the correspondence of LGs and kLGs (Kimball’s linkage group) were identified as: LG1-kLG6, LG2-kLG1, LG3-kLG3, LG4-kLG9, LG5-kLG7, LG6-kLG4, LG7-kLG8, LG8-kLG5 and LG9-kLG2. (Table 1 appeared after this paragraph).

### Comparative genomics analysis among grasses

**Fig. 2:**
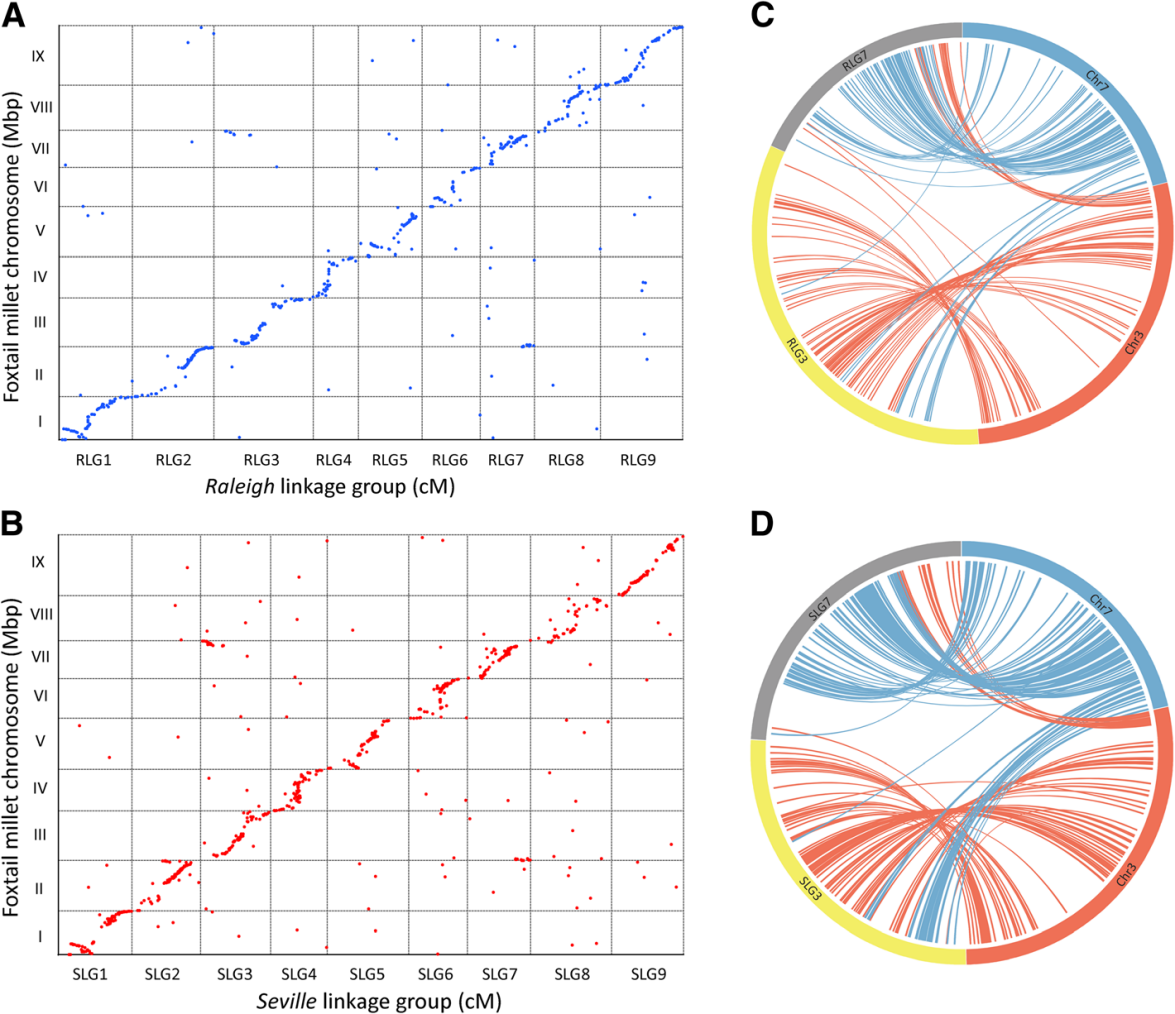
Genomic comparison between the St. Augustinegrass and foxtail millet genomes. **a**, **b** Dot-plot diagram showing synteny relationships between St. Augustinegrass LGs and foxtail millet chromosomes. Each dot represents a DNA marker. **c**, **d** Circos plot showing genome rearrangements between St. Augustinegrass LGs and foxtail millet chromosomes. RLG indicates Raleigh linkage group. SLG indicates Seville linkage group

Comparative genomics analysis between St. Augustinegrass and three other model grass species (foxtail millet, sorghum and rice) was performed to investigate syntenic conservation and chromosome rearrangements between them. Both St. Augustinegrass and foxtail millets belong to the Panicoideae subfamily in the grass family, with an equivalent basic chromosome number of *x* = 9. The blastn search against the foxtail millet genome revealed both ‘Raleigh’ and ‘Seville’ LGs showed orthologous relationships with foxtail millet chromosomes. St. Augustinegrass LGs were numbered in order based on the orthology to foxtail millet chromosomes. Among 1100 SNPs on ‘Raleigh’ LGs, 603 SNPs (54.8%) could be positioned on foxtail millet chromosomes, 544 of which could be mapped on the orthologous chromosomes (Table 2 and Additional file 1: Table S6). Meanwhile, 873 (55.6%) SNPs mapped on ‘Seville’ LGs were located on foxtail millet chromosomes, 794 of which could be placed on orthologous chromosomes (Table 2 and Additional file 1: Table S7). In addition, dot-plot diagrams showed that there was high collinearity between the genomes of St. Augustinegrass and foxtail millet (Fig. 2a, b).

**Table 2:** Number of St. Augustinegrass markers that mapped to foxtail millet, sorghum and rice genomes

			Number of markers mapped to foxtail millet genome			Number of markers mapped to sorghum genome			Number of markers mapped to rice genome	
‘Raleigh’ LG	‘Seville’ LG	Foxtail millet chr.	‘Raleigh’	Seville’	Sorghum chr.	‘Raleigh’	‘Seville’	Rice chr.	‘Raleigh’	‘Seville’
RLG1	SLG1	ChrI	75 (70)	122 (118)	Chr4	26 (24)	51 (48)	Chr2	18 (17)	36 (32)
RLG2	SLG2	ChrII	79 (76)	103 (97)	Chr2	28 (24)	42 (40)	Chr9 + Chr7	17 (8 + 5)	30 (21 + 4)
RLG3	SLG3	ChrIII + ChrVII	81 (65 + 12)	120 (82 + 25)	Chr8 + Chr9	20 (7 + 10)	44 (11 + 26)	Chr12 + Chr5	15 (7 + 4)	34 (9 + 19)
RLG4	SLG4	ChrIV	44 (42)	73 (66)	Chr10	18 (17)	25 (22)	Chr6	8 (8)	10 (8)
RLG5	SLG5	ChrV	65 (59)	90 (84)	Chr3	19 (18)	38 (32)	Chr1	14 (14)	27 (22)
RLG6	SLG6	ChrVI	34 (30)	66 (54)	Chr7	10 (9)	29 (24)	Chr8	9 (5)	22 (15)
RLG7	SLG7	ChrVII + ChrIII	89 (67 + 15)	125 (103 + 18)	Chr6	33 (28)	41 (37)	Chr4	18 (14)	24 (21)
RLG8	SLG8	ChrVIII	57 (48)	65 (45)	Chr5	20 (15)	22 (18)	Chr11	12 (7)	15 (10)
RLG9	SLG9	ChrIX	68 (60)	109 (102)	Chr1	25 (20)	46 (38)	Chr3 + Chr10	17 (11 + 4)	22 (15 + 2)
Total			603 (544)	873 (794)		199 (172)	338 (296)		128 (104)	220 (178)

* Number in parentheses indicates markers that could be mapped to orthologous chromosomes

Despite the high degree of synteny and collinearity between St. Augustinegrass and foxtail millet, several chromosome rearrangements were observed that differentiate the two species. These inter-chromosomal rearrangements occurred between ChrIII and ChrVII to result in RLG3 and RLG7, and SLG3 and SLG7 (Fig. 2c, d). One end of RLG3 as well as SLG3 was orthologous with the end of ChrVII in foxtail millet, while one end of RLG7 and SLG7 was positioned on ChrIII (Fig. 2c, d). In addition, there were chromosome inversions that occurred near the ends of RLG1, RLG5, RLG6 and homologous SLG1, SLG5, SLG6 (Fig. 2a, b).

**Fig. 3:**
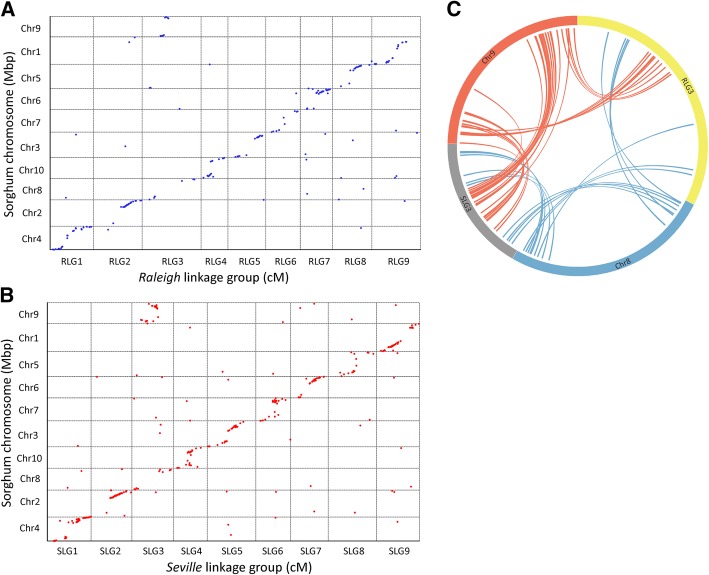
Genomic comparison between the St. Augustinegrass and sorghum genomes. **a**, **b** Dot-plot diagram showing synteny relationships between St. Augustinegrass LGs and sorghum chromosomes. Each dot represents a DNA marker. **c** Circos plot showing genome rearrangements between St. Augustinegrass LGs and sorghum chromosomes. RLG indicates Raleigh linkage group. SLG indicates Seville linkage group

Sorghum is another member of the Panicoideae subfamily, but with a higher basic chromosome number than St. Augustinegrass (*x* = 10). Comparative genomics analysis identified 199 and 338 SNPs on ‘Raleigh’ and ‘Seville’ LGs that could be located on sorghum chromosomes (Table 2, Additional file 1: Tables S8 and S9). Dot-plots showed high collinearity between genomes of St. Augustinegrass and sorghum (Fig. 3a, b). There was one to one correspondence for the orthologous chromosomes between St. Augustinegrass and sorghum, except that R(S)LG3 were orthologous with both Chr8 and Chr9 in sorghum (Fig. 3c). This relationship indicated that a nest chromosome fusion event occurred between Chr8 and Chr9 in sorghum to form R(S)LG3 in St. Augustinegrass.

**Fig. 4:**
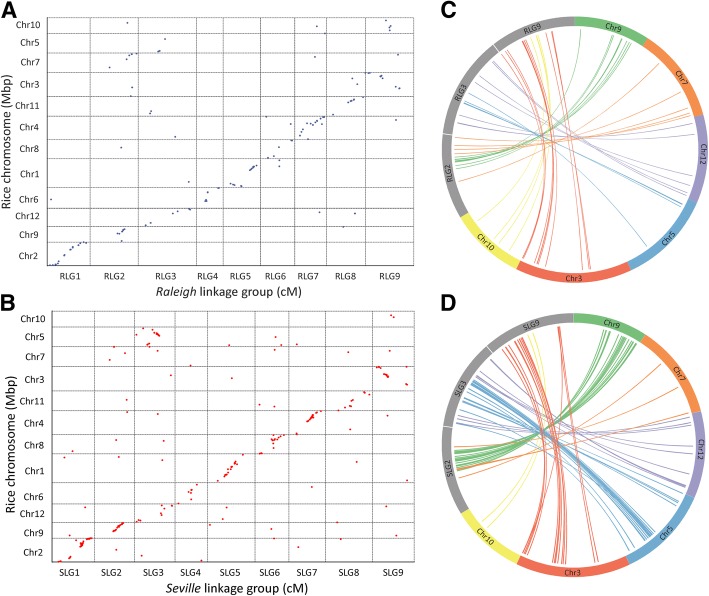
Genomic comparison between the St. Augustinegrass and rice genomes. **a**, **b** Dot-plot diagram showing synteny relationships between St. Augustinegrass LGs and rice chromosomes. Each dot represents a DNA marker. **c**, **d** Circos plot showing chromosome fusion between St. Augustinegrass LGs and rice chromosomes. RLG indicates Raleigh linkage group. SLG indicates Seville linkage group.

The rice genome (*Oryza sativa*) has been commonly used as reference comparison for genome analysis in the grass family as it has retained 12 basic chromosomes from the common ancestor of grass. There were 128 and 220 SNPs on ‘Raleigh’ and ‘Seville’ LGs that could be mapped on rice chromosomes (Table 2, Additional file 1: Tables S10 and S11). R(S)LG2, R(S)LG3 and R(S)LG9 were orthologous with rice Chr7 and Chr9, Chr12 and Chr5, Chr3 and Chr10, respectively. Rice Chr9, Chr5 and Chr10 were fused to the middle region of Chr7, Chr12 and Chr3 to form R(S)LG2, R(S)LG3 and R(S)LG9, respectively (Fig. 4c, d). These results suggested that three separate pairs of chromosomes fused to form three chromosomes in St. Augustinegrass during the evolution of the grass family. (Table 2 appeared after this paragraph).

### QTL identification for turf quality-related traits

Four turf quality-related traits (overall turf quality, leaf texture, genetic color, and turf density) evaluated under multiple environments in Kimball et al. were selected for QTL analysis [10]. A total of 48 candidate QTL were detected in all environments (Table 3). Among them, 11 QTL were identified for overall turf quality, 11 QTL for leaf texture, 16 QTL for genetic color and 10 QTL for turf density. All 48 QTL were distributed on all linkage groups with the exception of LG6 and LG7.

**Table 3:** Number and characteristics of QTL identified for turf quality traits under both individual and across environments

Trait^a^	Environment^b^	LG	Peak position (cM)	Interval (cM)^c^	Nearest marker	LOD^d^	Explained variance (%)^e^	A_f_^f^	A_m_^f^	D^f^
LT	Across	LG2	174.09	173.09–176.09	SSR16939	4.22 (3.90)	16.50	0.1401	0.2081	0.1172
		LG3	38.27	38.27	SSR3677	4.66 (3.90)	18.00	−0.2001	0.0028	0.1264
		LG3	54.93	54.93	SNP33683	3.94 (3.90)	15.50	−0.0540	− 0.2100	0.0819
		LG5	90.54	90.54	SSR14922	4.49 (3.90)	17.40	0.1776	0.1610	0.1160
LT	LW2013	LG8	52.79	52.79	SSR4549	4.86 (3.90)	18.70	−0.1607	− 0.2487	0.0118
LT	LS2013	LG2	80.90	72.61–80.90	SSR2502	4.25 (3.90)	16.60	1.3367	1.1968	−1.1999
		LG2	122.33	111.13–131.80	SNP41141	4.45 (3.90)	17.30	0.2548	0.0976	−0.1621
		LG2	162.83	162.83	SNP8925	4.12 (3.90)	16.10	−0.0877	0.3335	−0.0271
		LG3	38.27	38.27	SSR3677	4.61 (3.90)	17.90	−0.2474	0.0275	0.1660
		LG5	90.54	90.54	SSR14922	4.49 (3.90)	17.40	1.2973	1.1994	−1.2529
LT	LW2015	LG3	47.03	47.03–54.94	SNP49855	3.98 (3.90)	15.60	−0.0363	− 0.2124	0.1925
TQ	Across	LG3	38.27	37.55–40.55	SSR3677	5.27 (3.80)	20.10	−0.2451	−0.0453	0.1486
		LG3	53.57	53.57	SSR27743	5.60 (3.80)	21.20	−0.2276	−0.0420	0.2049
		LG8	40.44	37.26–42.44	SNP48740	6.14 (3.80)	23.00	−0.3092	−0.2039	0.0069
TQ	LW2013	LG3	38.27	38.27	SSR3677	3.83 (3.80)	15.10	−0.2217	0.0234	0.1623
		LG3	53.57	53.57	SSR27743	4.24 (3.80)	16.50	−0.2159	0.0178	0.2231
		LG3	79.76	79.76	SSR4343	4.66 (3.80)	18.00	−0.3087	−0.0263	−0.1565
TQ	LS2013	LG3	38.12	35.66–38.97	SNP41416	5.07 (3.80)	19.40	−0.2549	−0.0424	0.2803
		LG8	39.44	38.08–40.44	SNP48740	4.34 (3.80)	16.90	−0.3661	−0.1268	0.0909
TQ	LW2015	LG8	40.44	36.71–45.4	SNP48740	5.76 (3.90)	21.80	−0.3047	−0.2754	− 0.0340
		LG8	49.92	48.04–50.27	SNP15495	4.62 (3.90)	17.90	−0.1179	−0.2903	− 0.0439
		LG8	53.79	53.80–53.83	SSR4549	4.11 (3.90)	16.10	−0.1321	−0.3470	− 0.0941
GC	Across	LG5	74.82	74.24–74.82	SNP54683	4.58 (3.90)	17.70	0.2695	−0.0548	0.1544
		LG5	81.83	81.61–82.01	SNP35732	4.79 (3.90)	18.50	0.2814	−0.0202	0.2095
		LG8	41.44	36.13–50.27	SNP48740	6.23 (3.90)	23.30	−0.2638	−0.2916	− 0.0944
GC	LW2013	LG1	102.07	100.40–102.08	SNP19531	4.30 (3.90)	16.80	0.0070	−0.3357	0.2061
		LG5	81.89	74.82–82.09	SNP61660	4.35 (3.90)	16.90	0.3767	0.0233	0.1659
		LG8	42.44	41.44–42.44	SNP27553	4.01 (3.90)	15.70	−0.2456	−0.3268	−0.1035
GC	LS2013	LG2	167.00	165.83–170.09	SSR17849	4.78 (4.00)	18.40	−0.2383	0.1909	0.0964
		LG2	104.65	104.65	SNP32405	4.03 (4.00)	15.80	0.0887	−0.1759	0.1056
		LG3	68.23	68.23–68.24	SNP47138	4.13 (4.00)	16.10	−0.0678	−0.2228	− 0.0162
		LG3	75.23	75.23–76.33	SNP57077	4.69 (4.00)	18.10	0.1176	−0.2174	0.1139
		LG3	87.80	87.80	SNP32773	6.64 (4.00)	24.70	−0.1995	0.2065	0.2110
		LG5	71.77	62.44–74.24	SNP58680	4.59 (4.00)	17.80	0.2795	−0.3020	0.1802
		LG8	38.08	38.08–42.44	SSR4381	5.12 (4.00)	19.60	−0.1583	−0.0569	− 0.1734
		LG9	95.48	90.12–97.79	SNP21678	4.88 (4.00)	18.80	0.1195	−0.2410	−0.0226
GC	LW2015	LG1	102.07	102.07–102.08	SNP19531	4.09 (3.90)	16.00	−0.0289	−0.3180	0.2213
		LG8	41.44	32.50–50.27	SNP48740	5.64 (3.90)	21.40	−0.3007	−0.4067	− 0.0704
TD	Across	LG3	38.27	37.55–40.15	SSR3677	4.94 (3.60)	19.00	−0.3769	−0.1477	0.0900
		LG3	53.57	53.57	SSR27743	4.80 (3.60)	18.50	−0.3396	−0.0607	0.2076
		LG4	56.52	45.22–56.52	SNP5638	4.11 (3.60)	16.10	0.1477	0.3091	0.0777
		LG8	38.08	38.08	SSR4381	4.45 (3.60)	17.30	−0.2515	−0.1588	−0.1873
		LG8	56.38	56.38–56.62	SNP38207	4.20 (3.60)	16.40	−0.0725	−0.2320	− 0.3113
TD	LW2013	LG3	53.57	53.57	SSR27743	3.86 (3.80)	15.20	−0.3145	−0.0217	0.2322
TD	LS2013	LG3	38.39	28.80–40.55	SNP11022	5.82 (3.70)	22.00	−0.3024	−0.2121	0.3327
		LG3	53.57	53.57	SSR27743	4.29 (3.70)	16.70	−0.3753	−0.1512	0.2056
		LG4	45.84	39.32–56.52	SNP44515	4.23 (3.70)	16.50	0.2514	0.4000	−0.0119
		LG8	38.31	38.08–40.44	SNP7787	4.69 (3.70)	18.10	−0.4282	−0.2314	− 0.0490

^a^ Turf quality related traits evaluated in this study. LT = leaf texture; TQ = turf quality; GC = genetic color; TD = turf density

^b^ From Kimball et al. (2018): LW 2013 = Raleigh, NC in 2013, LW 2015 = Raleigh, NC in 2015, LS 2013 = Laurel Springs, NC in 2013, Across = combined analysis across environments

^c^ Regions with a LOD score above threshold values were considered as potential QTL interval

^d^ Genome-wide LOD threshold in parentheses

^e^ Percentage of phenotypic variation explained by the QTL

^f^ Allelic effects were estimated as A_f_ = [(μ_ac_ + μ_ad_) - (μ_bc_ + μ_bd_)]/4 for female (Raleigh) additivity; A_m_ = [(μ_ac_ + μ_bc_) - (μ_ad_ + μ_bd_)]/4 for male (Seville) additivity and D = [(μ_ac_ + μ_bd_) - (μ_ad_ + μ_bc_)]/4 for dominance where μ_ac_, μ_ad_, μ_bc_ and μ_bd_ are estimated phenotypic means associated to each of the 4 possible genotypic classes ac, bc, ad and bd, deriving for an ab × cd cross. Positive value indicates alleles increase the trait value

Several QTL were identified repetitively in different environments. For overall turf quality, position 38.27 cM on LG3 was confirmed in LW2013, LS2013 and Across, while 53.57 cM on LG3 was detected both in LW2013 and Across (Table 3). Another overlap region for overall turf quality was identified on 40.44 cM of LG8 in LS2013, LW2015 and Across. For genetic color, the 41.44–42.44 cM interval on LG8 was repetitively confirmed on all environments, while the 81.61–82.01 cM interval on LG5 was detected both in LW2013 and Across (Table 3). Four overlapping regions were identified for turf density, including 53.57 cM and 37.55–40.15 cM on LG3, 45.22–56.52 cM on LG4 and 38.08 cM on LG8 (Table 3). For leaf texture, three overlapping regions were found on LG3 (38.27 cM and 54.94 cM) and LG5 (90.54 cM) (Table 3).

In addition, we found QTL for different traits that co-located to the same region, especially on LG3 and LG8. There were seven QTL that overlapped in the interval of 35.66–40.15 cM on LG3, which included overall turf quality, turf density and leaf texture. On the same linkage group, there was another hot spot region (47.03–54.93 cM) that contained seven QTL for overall turf quality, turf density and leaf texture (Table 3). On LG8, 10 QTL for overall turf quality, turf density and genetic color overlapped in the 38.08–50.27 cM region (Table 3).

Sequences of markers within QTL regions were subsequently used for gene annotation analysis. The results showed several genes related to leaf formation and development, including: leaf trichome morphogenesis, anthocyanin biosynthetic, leaf senescence, auxin biosynthesis, cell wall metabolism, and wax/lipid biosynthesis (Additional file 1: Table S12) were included in these regions. The gene ontology (GO) analysis of these genes suggested a possible association between these genes and turf leaf morphology related traits. (Table 3 appeared after this paragraph).

## Discussion

### High density genetic map for St. Augustinegrass

Breeding efforts have been made to improve turf quality and its tolerance to biotic and abiotic stresses. Although conventional breeding methods are used in most turfgrass breeding programs, molecular breeding methods such MAS are becoming increasingly popular [17, 20]. MAS relies on marker-trait associations, hence high density genetic maps containing an abundance of molecular markers will maximize our ability to detect these associations. The St. Augustinegrass linkage map previously generated by Kimball et al. consisted of only 160 SSR markers with an average distance of 8.2 cM between markers [10]. In this study, we constructed an integrated linkage map containing 2871 SNP and 81 SSR markers with nine linkage groups. The map spanned 1241.7 cM, with an average distance of 0.4 cM between markers (Table 1). This map highly improved marker density and thus represents the densest genetic map for St. Augustinegrass to date. Furthermore, our map also integrates two types of molecular markers, SNPs and SSRs. While SNP-based high density linkage maps have been successfully used for comparative genomics analysis and QTL mapping in turfgrass [17, 21], SSR markers usually provide high levels of polymorphism information. However, SSRs are more labor intensive while SNP markers are highly abundant and high throughput. Thus, high density genetic maps that include both marker types are advantageous and can be very informative for comparative genomics and QTL analyses. Linkage maps with both SNP and SSR markers have been reported in many species such as pear and wheat [22, 23].

### Comparative genomics study

The grass family is arguably the most important family in agriculture. It provides abundant resources for plant evolutionary studies due to the presence of variation in basic chromosome numbers and a high frequency of polyploidy [24–29]. The advancement of genomic information available for several grass species, such as sorghum, wheat, maize and rice, has promoted numerous comparative genomics studies among grass family members [24–26]. It has been accepted that grass genomes have evolved from a common ancestor which underwent a series of whole-genome duplications, chromosome fusions and rearrangements to produce an intermediate ancestor with 12 basic chromosomes, although there is still argument on the base chromosome number of this common ancestor (*x* = 5 or *x* = 7) [27–29]. This presumed 12-chromosomes intermediate ancestor had a very similar chromosome arrangement with current-day rice (2*n* = 2*x* = 24). Most grass genomes are hypothesized to have formed from this intermediate ancestor through chromosome fusions, leading to reduction in chromosome numbers and additional rearrangements [29].

Panicoideae has a predominant base chromosome numbers of *x* = 9 and *x* = 10 [6]. It was previously hypothesized that nested chromosome fusion (NCF) is the dominant mechanism for reduction of chromosome numbers in the grass family [25, 26]. Panicoideae ancestral genomes with *x* = 9 and *x* = 10 may have evolved from the *x* = 12 intermediate ancestor through three and two, respectively, NCFs. Comparative genomic analysis between sorghum (*x* = 10) and rice (*x* = 12) determined that sorghum chromosome Sb 1 originated from the insertion of the entire rice chromosome Os 10 to the centromeric region of Os 3, while Sb 2 was formed by insertion of Os 9 into Os 7 [25]. In addition, comparison between foxtail millet (x = 9) and rice found that foxtail millet chromosomes 2, 3 and 9 were collinear with rice chromosomes 7 and 9, 5 and 12, and 3 and 10 respectively, which indicates that another single NCF occurred in the evolution of the foxtail millet genome in addition to the two NCFs that happened in sorghum [30]. In our study, we found that St. Augustinegrass R(S)LG2, R(S)LG3 and R(S)LG9 were orthologous with rice Chr7 and Chr9, Chr12 and Chr5, Chr3 and Chr10, respectively. The rice Chr9, Chr5 and Chr10 were fused to the middle region of Chr7, Chr12 and Chr3 to form R(S)LG2, R(S)LG3 and R(S)LG9 (Fig. 4). Consistent with previous hypotheses, our results suggest that the St. Augustinegrass genome has evolved from the intermediate ancestor through three NCFs. By comparing the genomes of sorghum and foxtail millet, Zhang et al. found that NCF fused chromosomes 8 and 9 of sorghum in chromosome 3 of foxtail millet [30]. Similar results were observed in the present study, where St. Augustinegrass R(S)LG3 were found to be orthologous with both Chr8 and Chr9 in sorghum (Fig. 3). These results indicated that this NCF event most likely occurred before the divergence of foxtail millet and St. Augustinegrass. The high degree of synteny and collinearity between St. Augustinegrass and foxtail millet observed in our results indicates a very close evolutionary relationship between the two species (Fig. 2a, b). However, there were inter-chromosomal rearrangements between St. Augustinegrass LG3 and LG7 and foxtail millet chromosomes 3 and 7 (Fig. 2c, d). Such chromosome rearrangement events might have introduced genetic variation and contributed to divergence between these species.

### QTL identification for turf quality related traits

The high density linkage map generated in this study provided a platform for mapping QTL associated with traits of agronomic importance in St. Augustinegrass. Turfgrass quality is defined as the degree to which a turf conforms to an agreed upon standard. The components of turfgrass quality adopted by NTEP include uniformity, shoot density, leaf texture, leaf orientation, smoothness, and color (NTEP, 2017). In the present study, leaf texture, turf density, genetic color and overall turf quality were selected to evaluate the aesthetic performance of St. Augustinegrass. Kimball et al. detected eight QTL distributed on four LGs for these traits using an SSR-based linkage map [10]. In the present study, 48 putative QTL regions associated with these traits were successfully identified. These QTL regions were distributed on seven of nine LGs (Table 3). The detection power and resolution of QTL mapping was significantly improved by the high density linkage map compared to previous SSR-based map. Among these QTL, a number of occurrences of overlapping QTL for leaf texture, turf density, genetic color and overall turf quality were observed on LG3 (35.66–40.15 cM and 47.03–54.93 cM) and LG8 (38.08–50.27 cM) (Table 3). Co-location of QTL for different traits may indicate common genetic mechanisms for these traits, suggesting the importance of these regions for fine mapping as well as MAS [31]. Overlapping of QTL might also indicate that these regions contain genes controlling development and morphology of leaves and shoots. By blasting marker sequences within these regions, several orthologous genes associated with leaf formation and development, including: leaf trichome morphogenesis, anthocyanin biosynthesis, leaf senescence, auxin biosynthesis, cell wall metabolism, and wax/lipid biosynthesis (Additional file 1: Table S12) were found. For example, the orthologous genes of *PNH1* in *Arabidopsis* (*PNH/ZLL*) and rice (*OsPNH1*) were both reported to play important roles in the formation of the shoot apical meristem (SAM) from where leaves are produced [32, 33]. *DTX,* which encodes detoxification proteins, also known as Multidrug and Toxic Compound Extrusion (MATE) transporters in plants have been reported to affect plant architecture through the auxin and ABA pathways [34, 35]. It is speculated that the identified QTL regions might be controlling the turf quality traits in part through the orthology of these genes on the genome of St.Augustinegrass. However, further experiments need to be implemented to verify our results and improve the scale and quality of putative QTL, and to identify functional genes controlling turf quality.

Further analysis of the QTL regions found here to be associated with turf quality may help elucidate the genetic mechanisms of these complex traits and improve our ability to select for them during breeding cycles. The high density St. Augustinegrass genetic map, the first of its kind of the species, has the potential to assist in the identification of marker-trait associations for numerous qualitative and quantitative traits of economic and agronomic importance, such as turf quality and tolerance to environmental stresses. These associations can be subsequently used in MAS and thus increase the efficiency of selection in St. Augustinegrass breeding.

## Conclusions

Overall, we identified thousands of SNP markers in St. Augustinegrass using a GBS approach and constructed a high density genetic map including both SNP and SSR markers. To date, this is the most comprehensive genetic map developed for this species. Using this genetic map, we conducted comparative genomics analysis between St. Augustinegrass and foxtail millet, sorghum and rice, which revealed chromosomal rearrangement events that occurred during the evolutionary history of the grass family. These results provide a genetic and genomic basis for future functional gene cloning and genome assembly. In addition, several turf quality-related QTL were identified, which were distributed on different linkage groups. The high density genetic map and identified QTL will enhance turfgrass improvement programs.

## Methods

### Plant materials and DNA extraction

A pseudo-F_2_ population consisting of 115 hybrids was derived from a cross between St. Augustinegrass cultivars ‘Raleigh’ and ‘Seville’ (both parents are diploids, 2*n* = 2*x* = 18) following artificial hybridization methods [10]. This population was obtained from North Carolina State University Center for Turfgrass Environmental Research and Education. Each individual was propagated vegetatively in plastic containers containing Fafard potting mix (Conrad Fafard Inc., Agawam, MA) and maintained in the greenhouse at North Carolina State University, Raleigh, NC, USA. Young leaves of each hybrid along with parents were collected for genomic DNA extraction. The quality of the DNA was first visualized by agarose gel electrophoresis and further tested using a NanoPhotometer (Implen, München, Germany). DNA concentration was quantified using a Hoefer DQ 300 fluorometer (Hoefer, Holliston, United States).

### GBS library construction

The sequencing library was prepared according to the procedure detailed in Poland et al. with minor modifications [36]. Approximately 200 ng of genomic DNA for each sample (115 hybrids and two parents) was digested with *PstI* and *MspI* (New England BioLabs, Inc.; Ipswich, MA) restriction enzymes for 2 h at 37 °C in a 20 μL volume. The reaction was stopped by incubation at 65 °C for 20 min. Barcoded adapters (containing unique barcode sequences, details in Additional file 1: Table S1) and a common-Y adapter were ligated to digested genomic DNA fragments at 22 °C overnight in 40 μL volume and stopped at 65 °C for 20 min. Then, 10 μL of each sample was pooled and cleaned up using QIAquick PCR Purification Kit (QIAGEN, Hilden, Germany). After that, purified DNA was amplified using NEB MasterMix (New England BioLabs, Inc.; Ipswich, MA). PCR products were purified and size-selected using GeneRead Size Selection Kit (QIAGEN, Hilden, Germany) to remove adapter dimers and small fragments (< 150 bp). The library was size-selected at a range of 250–400 bp using D1000 ScreenTape assay (Agilent, Waldbronn, Germany) and sequenced on Illumina HiSeq 2500 (Illumina, San Diego, United States).

### SNP identification and genotyping

In this study, the non-reference UNEAK pipeline was used to perform SNP discovery and genotyping [15, 37]. GBS raw reads were processed to keep only reads that contained barcodes and the restriction site. High quality reads were trimmed to 64 bp and identical reads were collapsed into tags. Pairwise alignment identified tag pairs with a single base pair mismatch, which could be considered as candidate SNPs. A network filter (Error tolerance rate = 0.03) was employed to discard repeats, paralogs and error tags. The remaining reciprocal tag pairs could then be identified as SNPs. Finally, the SNPs were filtered by sequencing depth (≥ 10), minor allele frequency (≤ 0.05) and call rate (≥ 90%) to obtain high quality SNPs.

### Linkage map construction

JoinMap 4.0 [19] was used to construct the linkage map. SNP markers were assigned to three categories according to segregation type: heterozygous in parent ‘Raleigh’ and homozygous in parent ‘Seville’ (‘lm x ll’ type), homozygous in parent ‘Raleigh’ and heterozygous in parent ‘Seville’ (‘nn x np’ type), heterozygous in both parents (‘hk x hk’ type). Markers that showed abnormal segregation ratios (chi-squared test, df = 2, cut off value = 9.21, *P* < 0.01) were excluded from map construction. The ‘lm x ll’ type and ‘nn x np’ type SNP markers were used to construct separate parental linkage maps for parent ‘Raleigh’ and ‘Seville’, respectively. Meanwhile, ‘hk x hk’ type SNP markers along with previously identified SSR markers from Kimball et al. [10], were used to integrate the parental linkage groups into a consensus map. All linkage maps were constructed using the regression mapping algorithm with a minimum LOD of 9.0 and a maximum recombination rate of 0.4 (goodness-of-fit jump value 3.0 and ripple value 1). Map distances were calculated using the Kosambi mapping function. The map quality was checked with ‘N.N. fit’ function in JoinMap 4.0. MapChart 2.32 was used to visualize the linkage maps [38].

### Comparative genomics analysis

The sequences of mapped SNP tags were aligned to genome sequences of model grass species: foxtail millet (*Setaria italica*), sorghum (*Sorghum bicolor*) and rice (*Oryza sativa*) using the blastn program in BLAST+ 2.6.0 [39] with an e-value cutoff of 1 × 10^− 5^. Reference genomes *Setaria italic* v2.0, *Sorghum bicolor* NCBIv3, *Oryza sativa* Japonica Build 4.0 were downloaded from the NCBI genome database. Marker sequences that showed hits to reference genomes were used for further comparative analysis. Comparative results were visualized using the dot-plot in R package ggplot2 and Circos plot in Circos package [40, 41].

### QTL mapping of turf quality-related traits

Turf quality-related traits evaluated by Kimball et al. were used for QTL mapping [10]. All hybrids together with parental lines were planted in a randomized completed block design (RCBD) with three replications at two locations (Raleigh and Laurel Springs, NC, United States) and evaluated for two years (2013 and 2015). Turf quality-related traits, including overall turf quality, leaf texture, genetic color, and turf density were evaluated visually on a 1 to 9 scale according to the National Turfgrass Evaluation Program’s (NTEP) guidelines as follows: turf quality, 1 = poor quality and 9 = excellent quality; leaf texture, 1 = coarsest texture and 9 = finest texture; genetic color, 1 = light green/yellow and 9 = dark green; turf density, 1 = sparsest density and 9 = densest turf. Each year by each location combination was considered as a separate environment. An analysis of variance (ANOVA) and least square (LS) means were generated using the GLM procedure in SAS statistical software version 9.4 (SAS Inst. Inc., 2017) for each trait. QTL analysis was performed using LS mean values both for individual environments and across environments against the integrated linkage map using MapQTL 6.0 [42]. Interval mapping (IM) and multiple QTL method (MQM) analysis were performed to detect significant associations between markers and phenotypic traits using a regression approach. LOD thresholds (*P* < 0.05) for genome-wide were determined for each trait using a permutation test with 10,000 iterations. Regions with a LOD score above threshold values were considered as potential QTL intervals. Allelic effects were estimated as A_f_ = [(μ_ac_ + μ_ad_) - (μ_bc_ + μ_bd_)]/4 for female (Raleigh) additivity; A_m_ = [(μ_ac_ + μ_bc_) - (μ_ad_ + μ_bd_)]/4 for male (Seville) additivity and D = [(μ_ac_ + μ_bd_) - (μ_ad_ + μ_bc_)]/4 for dominance where μ_ac_, μ_ad_, μ_bc_ and μ_bd_ are estimated phenotypic means associated to each of the 4 possible genotypic classes ac, bc, ad and bd, deriving for an ab × cd cross [43]. Furthermore, sequences of markers within the identified regions of interest were searched against the NCBI NR database using blastn/blastp tools to obtain their orthologs. Gene Ontologoy (GO) annotation was conducted using UniProt database to predict gene function in the QTL regions.

## Additional file

Additional file 1: **Table S1** to **S12**. **Table S1.** Sequence of barcodes and adapters in St. Augustinegrass GBS library. **Table S2.** Number of sequencing reads for St. Augustinegrass parent lines and hybrids. **Table S3.** Detail of linkage group and marker sequences of ‘Raleigh’ St. Augustinegrass genetic map. **Table S4.** Detail of linkage group and marker sequences of ‘Seville’ St. Augustinegrass genetic map. **Table S5.** Detail of linkage group and marker segregation in integrated St. Augustinegrass genetic map. **Table S6.** Genomics comparison between ‘Raleigh’ St. Augustinegrass linkage groups and foxtail millet genome. **Table S7.** Genomics comparison between ‘Seville’ St. Augustinegrass linkage groups and foxtail millet genome. **Table S8.** Genomics comparison between ‘Raleigh’ St. Augustinegrass linkage groups and sorghum genome. **Table S9.** Genomics comparison between ‘Seville’ St. Augustinegrass linkage groups and sorghum genome. **Table S10.** Genomics comparison between ‘Raleigh’ St. Augustinegrass linkage groups and rice genome. **Table S11.** Genomics comparison between ‘Seville’ St. Augustinegrass linkage groups and rice genome. **Table S12.** Gene ontology analysis of sequence within QTL regions related to leaf development. (XLSX 346 kb)

## Abbreviations

Chr: Chromosome
cM: Centimorgan
GBS: Genotyping by sequencing
GO: Gene ontology
GWAS: Genome-wide association study
IM: Interval mapping
LG: Linkage group
LOD: Logarithm of the odds
MAS: Marker-assisted selection
MQM: Multiple QTL method
NTEP: National Turfgrass Evaluation Program
QTL: Quantitative trait locus
SNP: Single nucleotide polymorphism
SSR: Simple sequence repeat
